# Vaccine Perceptions Outweigh Emotional Flow in Predicting HPV Vaccination Intentions Among Gen Z College Students

**DOI:** 10.3390/vaccines14020150

**Published:** 2026-02-03

**Authors:** Christopher Hominski, Carolyn A. Lin

**Affiliations:** Department of Communication, University of Connecticut, Storrs, CT 06269-1259, USA; christopher.hominski@uconn.edu

**Keywords:** Extended Parallel Process Model, Emotion and Adaptation Theory, emotional flow, risk-taking tendencies, HPV vaccines, vaccine perceptions, perceived vaccine effectiveness, HPV prevention message sources

## Abstract

Background/Objectives: HPV vaccination rates among U.S. young adults remain unchanged at 47% since 2019. Barriers including misinformation, vaccine hesitancy, and stigma surrounding HPV’s long-standing association with sexually transmitted infections have limited widespread acceptance among the male population. This experimental study explores how prevention messages incorporating an emotional flow element may influence vaccination intention. It also examines whether vaccination status may differentiate pre-exposure risk-taking tendencies and vaccine perceptions—as well as post-exposure HPV susceptibility, HPV severity, vaccine effectiveness, and emotional response—among young adults. Methods: A one-factor between-subjects experiment (including facts-only vs. facts→threat vs. facts→threat→hope conditions) was conducted online with a group of Gen Z college students at a U.S. university (*N* = 440). Results: ANCOVA results indicated that emotional flow embedded in the three message conditions did not result in significantly different emotional responses (across all participants) or vaccination intention among the unvaccinated participants. Whereas vaccinated participants reported greater perceived vaccine benefits, HPV susceptibility, HPV severity, and vaccine effectiveness, unvaccinated participants exhibited stronger emotional responses toward the facts→threat→hope message instead. Regression results revealed that vaccine perceptions, risk-taking tendencies, HPV susceptibility, and emotional response significantly predicted vaccination intention, in that order. TV advertising was identified as the leading HPV information source, followed by social media advertisements and recommendations from health professionals. Conclusions: These findings highlight that incorporating emotional flow may enhance message engagement among unvaccinated individuals. HPV campaigns should consider increasing positive vaccine perceptions, alleviating perceived threat of HPV, and eliciting positive emotional response toward vaccination acceptance and adoption.

## 1. Introduction

Human papillomavirus (HPV) is a major public health concern linked to cervical, throat, anal, and penile cancer risks, in addition to genital warts, which is a more immediate outcome for many young adults. Although highly effective vaccines are widely available, vaccination rates of one dose or more among U.S. young adults aged 18 to 26 years (who were not vaccinated as a minor) did not exceed 47% since 2019 [1]. Barriers such as misinformation, vaccine hesitancy, and stigma surrounding HPV’s long-standing association with sexually transmitted infections (STIs) have limited widespread acceptance among the male population [2]. These challenges were further compounded during the COVID-19 pandemic, as healthcare professionals reported increased HPV vaccine hesitancy and refusal linked to broader vaccine mistrust and misinformation in the U.S. [3].

Within the U.S. healthcare system, HPV vaccination is typically delivered through primary care clinics, pediatric and family medicine practices, pharmacies (in many states), college health services, and public health clinics. Vaccination cost coverage varies by health insurance status and eligibility for publicly funded programs; access barriers can thus include insurance coverage and whether a trusted clinician recommends vaccination. The vaccine is routinely recommended by healthcare providers to parents for their early adolescents, with catch-up vaccination recommended through young adulthood.

The initial 2006 HPV health campaigns in the U.S. primarily focused on raising awareness of HPV as a sexually transmitted infection (STI) linked to cervical cancer. This framing inadvertently introduced stigma, particularly among parents, who viewed vaccinating their children against HPV as implicitly endorsing adolescent sexual activity [4,5]. Over time, messaging about the HPV vaccine shifted from emphasizing HPV as an STI to highlighting cancer prevention, as reflected in national efforts such as the Centers for Disease Control and Prevention’s “HPV Is Cancer Prevention” campaign [6]. Concurrently, campaigns broadened their focus to include the risks of anal, penile, and oropharyngeal cancers and to explicitly target males [7].

The Extended Parallel Process Model [8] is a widely applied framework for explaining the effects of fear appeals. Prior HPV vaccination studies have utilized fear appeals by emphasizing the risk of HPV-associated cancers and the serious consequences of infection [9]. While fear-based messaging can be effective in gaining attention and awareness, prolonged exposure to fear messaging may trigger defensive avoidance or maladaptive coping behavior among those with low self-efficacy or low response efficacy [8,10]. Emotional flow, a construct that proposes utilizing specific message designs to shift an individual’s emotional state from negative to positive, has been validated as an approach that could help direct audiences away from fear to increase their efficacy in adaptive coping [11].

By integrating the constructs of EPPM and emotional flow, this study aims to examine whether HPV vaccination messages that display a shifting emotional tone with increased valence may help facilitate emotional response toward HPV and motivate vaccination intention. The current study contributes new insights to the literature through testing a proposed conceptual framework with a novel message strategy that may sustain audience attention, reinforce adaptive efficacy, and reduce defensive avoidance toward HPV prevention.

## 2. Literature Review

### 2.1. Message Appeals and Efficacy Perceptions

Fear appeals have long been a strategy ubiquitous to health communication, defined as messages that elicit fear by presenting a significant threat and recommending effective actions to mitigate it. Early models, such as the Drive Model, laid the foundation for understanding how fear motivates behavior [12]. The Extended Parallel Process Model (EPPM) is a typology that integrates the Parallel Process Model and Protection Motivation Theory to explain how individuals may respond to fear appeals [13,14,15]. EPPM proposes that while high perceived threat coupled with high efficacy can facilitate adaptive responses or danger control, high perceived threat combined with low perceived efficacy (response efficacy and self-efficacy) can lead to maladaptive responses or fear control [8,10].

Adaptive or danger-control responses may occur when individuals focus on managing the threat itself (e.g., seeking information, forming intentions to vaccinate, or engaging in other protective behaviors). Maladaptive or fear-control responses may arise when individuals focus on managing their emotional reaction to the threat rather than the threat itself (e.g., avoidance, message rejection, minimization, or defensive processing). Thus, threat-based messages are most likely to produce adaptive outcomes when they are accompanied by clear, credible efficacy information that strengthens response efficacy and self-efficacy, thereby supporting danger-control rather than fear-control processing.

McGlone et al. demonstrated that HPV vaccination messages were most persuasive when perceived threat was paired with strong efficacy information, such as clear evidence of vaccine effectiveness (i.e., response efficacy) and recommendations from trusted health authorities [16]. Limbu and Huhmann found that fear appeals elicited adaptive danger-control responses only when accompanied by strong efficacy cues, whereas fear-based messages without efficacy support increased maladaptive fear-control processing [17].

Gore and Campanella Bracken reported qualified support for EPPM, showing that a meningitis risk message combining low (instead of high) threat with high efficacy elicited adaptive danger-control responses, whereas a high threat message without (instead of low) efficacy support prompted maladaptive fear-control responses [18]. Popova challenges the assumption that perceived efficacy solely determines whether individuals engage in fear- or danger-control responses, noting the lack of unequivocal empirical support for the full set of EPPM’s 12 core propositions [19]. Popova further argues factors such as anxiety and sensation seeking may have direct effects on behavioral outcomes, independent of the EPPM pathways [19].

### 2.2. Message Appeals and Emotional Flow

As a fear appeal containing the element of threat and efficacy is considered a potentially effective messaging strategy to facilitate protection motivation [8], this suggests that fear may transition into hope as an adaptive coping response. For instance, based on Lazarus’ theory of emotion and adaptation, Xu and Lin suggest that hopeful feelings could rise as an adaptive response to fearful feelings to ameliorate a threat, when offered a relief stimulus (e.g., COVID-19 vaccine) [20,21]. Lazarus’ emotional adaptation theory was further conceptualized by Nabi’s proposition, which asserts that discrete emotions could stimulate a cognitive response to seek relief from negative emotions to reinforce positive emotions [20,22].

Building on earlier theorizing about discrete emotional responses, Nabi introduced the concept of emotional flow in persuasive health messages, describing how sequences of emotional shifts during message exposure can influence attention, engagement, and persuasion [23]. Nabi and Green further proposed the construct of emotional flow, which describes the evolution of emotions during message exposure when messages can shift emotions from fear to hope to sustain audience engagement and enhance the message’s persuasive outcomes [11]. Alam and So maintain that messages shifting from fear to reassurance were more persuasive than those that rely on fear alone [24]. Fitzgerald et al. validated the Emotional Flow Scale (EFS) and showed that messages with greater emotional variability, especially negative-to-positive (N-P) shifts, elicited stronger cognitive elaboration and interpersonal discussion when compared to single-valence messages [25].

Peinado and Nabi manipulated and tested the effect of deliberate transitions from negative emotions (e.g., fear or concern) to positive emotions (e.g., hope or empowerment) [26]. Their findings indicated that Negative-to-Positive messages led to higher perceived self-efficacy, stronger protection behavioral intentions, and greater interpersonal “talk” about the message, compared to messages that stayed consistently fear based. Their work demonstrates that concluding a message on a positive emotion (like hope) may leave audiences feeling empowered rather than overwhelmed, which is particularly valuable in health contexts where fear appeals may backfire. By implication, based on the fundamental assumptions of emotional adaptation and emotional flow, utilizing a message to transition negative emotions toward HPV to positive emotions toward vaccination effectiveness may mitigate a potential defensive response toward the fear to increase adaptive coping [20,22].

To further validate the existing literature, we will extend prior work by adding a third condition, which presents only the facts, to serve as a baseline for comparison. Furthermore, as only about a handful of studies have examined the cognitive and/or affective responses toward HPV between vaccinated and unvaccinated individuals, the current research will compare these two groups to help us understand the potentially effective or ineffective messaging strategies. A discussion of the relevant literature is provided below.

*Vaccinated* vs. *Unvaccinated Groups*. Based on a Brazilian interview study with adolescents 11–19 years old, unvaccinated adolescents were less knowledgeable about HPV and HPV vaccines, compared to their vaccinated counterparts [27]. These findings were confirmed by a U.S. adolescent survey (aged 14–19) conducted in a Colorado school, which indicated a similar knowledge gap between vaccinated and unvaccinated groups, including the beliefs that only women were subject to HPV and HPV vaccination was recommended for females aged 11–26 [28]. Similar results were also reported by a U.S. online survey with cisgender HIV-negative women (aged 18–45) conducted in the city of Miami, which further evidenced that unvaccinated individuals were less knowledgeable about their HPV risk and infection prevention [29].

As for young adult studies, an online survey conducted in the Netherlands with males (aged 19–26) suggested that unvaccinated men reported lower perceptions of HPV severity and vaccine benefits—as well as greater vaccination barriers—relative to vaccinated men [30]. These findings were validated by a U.S. online survey conducted with college students (aged 18–26) in Tennessee, which revealed that the vaccinated students perceived lower vaccination barriers as well as a higher level of HPV knowledge and perceived HPV risk, compared to unvaccinated students [31].

Accordingly, our hypotheses will reflect expected differences in message processing associated with prior vaccination status, rather than causal effects of message exposure on prior vaccination behavior. These hypotheses will explore an emotional shift structure to show how a message with only scientific facts vs. a message with facts and threat vs. a message with facts, threat, and hope may elicit a differential emotional response between the vaccinated vs. unvaccinated individuals.

**H1a,b.**  *Exposure to the facts→threat→hope message will generate stronger positive emotional responses than exposure to (a) the facts→threat message and (b) the facts-only message among vaccinated than unvaccinated individuals.*

**H1c.**  *Exposure to the facts→threat message will generate stronger negative emotional responses than exposure to the facts-only message among vaccinated than unvaccinated individuals.*

### 2.3. Message Appeals and Vaccination Intention

When people perceive a health threat as serious and personally relevant, they are more likely to take action to protect themselves. Research has shown that highlighting the severe consequences of HPV, such as cervical and throat cancer, increases motivation to vaccinate [32]. In addition, HPV message-framing research suggests that how HPV is framed emotionally (e.g., shame vs. efficacy-affirming frames) can shift vaccination intentions, underscoring the importance of appeal strategy beyond simply presenting risk [33]. In particular, fear appeals tend to be most effective when they emphasize the long-term risks of inaction [10].

Perceptions of HPV vaccine messaging can play a crucial role in shaping vaccine uptake. For instance, Chen et al. found that high-severity messages on social media significantly increased vaccination intentions, especially when paired with information about the vaccine’s effectiveness [8]. Gilkey et al. examined parents’ views on the most and least persuasive reasons for HPV vaccination and found that cancer prevention was the most compelling argument [34]. Their research further suggests that while fear-based appeals can be effective, messages that emphasize long-term benefits and safety may better resonate with hesitant parents. By implication, integrating positive emotional transitions into health narratives can enhance persuasion and engagement.

Carcioppolo et al. similarly reported stronger vaccination intentions when messages balanced threat content with efficacy cues [35]. They found that messages with a one-on-one threat-to-efficacy ratio (1:1) were most effective in promoting vaccination intentions among young women. Fitzgerald et al. further confirmed that negative-to-positive (N-P) emotional shifts could generate enhanced cognitive elaboration to promote adaptive coping [25]. Likewise, the approach of Negative-to-Positive messaging was also shown to heighten stronger behavioral intentions toward health protection behavior [26].

Based on the theoretical reasoning and empirical findings elucidated above, the hypotheses below will test the effects of emotional shifts in messaging on HPV vaccination intentions on unvaccinated individuals.

**H2a,b.**  *Exposure to the facts→threat→hope message will generate a stronger vaccination intention than exposure to (a) the facts→threat message and (b) the facts-only message.*

**H2c.**  *Exposure to the facts→threat message will generate a stronger vaccination intention than exposure to the facts-only message.*

### 2.4. Risk-Taking Tendency and Vaccine Perceptions

Risk-taking behavior is a critical factor in understanding health decision-making, particularly in the context of preventive measures such as HPV vaccination. Individuals who engage in frequent risk-taking behaviors may perceive health threats differently than those that are more risk-averse, either through underestimating their risk susceptibility (due to optimistic bias) or recognizing a heightened risk due to their own behaviors [36]. Brewer and Fazekas found that those who engaged in frequent risk behaviors, such as unprotected sex or multiple sexual partners, were more likely to perceive HPV as a personal threat but were not necessarily more likely to be vaccinated [36]. This suggests an attitude–behavior gap where perceived risk susceptibility may be high, but other factors such as necessity perception, vaccine safety, or mistrust in healthcare systems may still serve as barriers to vaccine uptake.

Given that HPV vaccine uptake has remained stagnant despite strong public health campaigns, understanding the role of vaccine hesitancy is essential for designing more effective, targeted messaging strategies [1]. To systematically measure vaccine hesitancy, the World Health Organization (WHO) Strategic Advisory Group of Experts (SAGE) on Immunization developed the Vaccine Hesitancy Scale (VHS) in 2015. This scale assesses individuals’ attitudes toward vaccines, including concerns about safety, effectiveness, and trust in health authorities, via perceived confidence and risk perception [37].

Studies using the VHS have found that concerns about vaccine safety and necessity are particularly relevant to HPV vaccine decision-making, suggesting that addressing these concerns directly may help increase uptake [34,35]. To develop more effective HPV vaccination messages, it is useful to understand how risk-taking tendencies and vaccine perceptions (e.g., benefits, safety, and necessity) may influence HPV vaccine uptake among vaccinated and unvaccinated individuals. These queries are explored in the research questions posed below.

**RQ1a,b.**  *Will (a) risk-taking tendencies and (b) vaccine perceptions be more positive for vaccinated than unvaccinated individuals?*

### 2.5. Threat Appraisal, Emotional Flow, and Vaccine Effectiveness

Prior research found that vaccinated individuals generally perceived themselves as more susceptible to HPV prior to vaccination, as their decision to vaccinate often stemmed from recognizing their personal relevance of the threat [32]. In particular, previous studies have shown that those who were vaccinated tend to view vaccines as a necessary preventive measure, while unvaccinated individuals or those who avoided vaccination were more likely to harbor doubts about vaccine benefits [37,38]. These empirical results appear to conceptually align with past research on vaccine confidence, which highlights the perceived benefits and risk appraisal.

For instance, Xu and Lin’s study emphasized that while fear stemming from negative appraisal of COVID-19 vaccines was connected to perceived vaccination barriers, hope emanating from positive appraisal of perceived vaccine effectiveness was linked to perceived vaccination benefits [21]. Their findings are consistent with Nabi and Prestin’s contention addressing the role of hope and fear in impacting health behavior—as well as Adam et al.’s results illustrating how the relationships between risk appraisals and the discrete emotion of hope—may impact COVID-19 vaccine uptake [39,40]. These studies also align with prior fear-appeal research, which suggests that emotional reactions to threat are most likely to translate into protective action when efficacy beliefs (self-efficacy and response efficacy) are also high, linking emotional response with perceived efficacy in determining outcomes [10,41].

Hence, consistent vaccination adoption is likely linked to stronger positive attitudes toward perceived vaccine effectiveness and benefits. For example, prior research has indicated that past vaccination experience reinforces perceptions of perceived vaccine effectiveness to drive future vaccination behavior, with vaccinated individuals more likely to trust its protective benefits compared to those who remain hesitant or unsure [42].

Importantly, vaccinated individuals may perceive future health risks (e.g., contracting HPV) as more salient because they had already acknowledged the threat and taken action to mitigate it [40]. As such, they were more likely to demonstrate stronger emotional responses to HPV-related messaging because they have already acknowledged the threat and acted on their risk appraisal [40].

To validate the literature addressing emotional and cognitive responses toward protective health action and the affiliated perceived action efficacy between vaccinated and unvaccinated individuals in each study condition, the following research hypotheses are posited.

**H3a,d.**  *Perceived (a) emotional response, (b) HPV susceptibility, (c) HPV severity, and (d) vaccine effectiveness will be greater for vaccinated than unvaccinated individuals across facts-only, facts→threat, and facts→threat→hope conditions.*

### 2.6. HPV Message Exposure

Understanding where audiences encounter HPV vaccine messages is crucial for evaluating the effectiveness of communication strategies. Prior studies have found that the medium through which a health message is delivered (e.g., television, online ads, social media platforms) significantly influences both message recall and attitudes toward vaccination [8]. According to a national study of adults aged 18–65 (*N* = 3444), while HPV and vaccine awareness among young adults aged 18–26 (14.4%) was much lower than those aged 27–35 (43.4%) and 45–65 (42.2%), HPV awareness odds ratio was higher for those who used the Internet than those who relied on social media [37]. The same study also found that while Internet use could improve HPV awareness, the same was not true for social media use or information received from religious leaders.

Jo et al. analyzed the data (*N* = 2498) from the 2020 Health Information National Trends Survey (conducted by the U.S. National Cancer Institute) and revealed that social media use was not associated with either HPV awareness or HPV knowledge [43]. Mueller et al. recruited an age- and gender-stratified quota sample of Latinos 21 years or older (*N* = 1334) from low-income and uninsured Latino immigrant populations to study their HPV information sources. Their results indicated that aside from Spanish television and Internet, healthcare providers were the primary sources for HPV information [44]. In contrast, Lama et al.’s study analyzed the survey data (*N* = 2720) from the forementioned Health Information National Trends Survey (2017–2019); it provided the only empirical finding in the literature that reported social media as a relevant HPV information source among adults with children living in the household [45].

These findings underscore the value of mediated vaccine communication strategies to gain audience attention. As the existing literature only examined a narrow set of HPV message sources, the current study intends to evaluate the exposure frequency of a more comprehensive range of HPV message sources. By assessing the exposure to different message sources, we will be able to identify those sources that may be more effective in reaching our target audience. To explore these HPV message exposure activities, two research questions are posed below.

**RQ2.**  *Will the exposure level across HPV message sources differ between vaccinated and unvaccinated individuals?*

**RQ3.**  *Will the exposure level to the different individual HPV message sources be related to vaccine perceptions, emotional flow, perceived HPV susceptibility, perceived HPV severity, and perceived vaccine effectiveness?*

## 3. Materials and Methods

### 3.1. Method

The sample for the study was recruited from the undergraduate student population of a northeastern university in the U.S., with prior IRB approval. Students received course credit in return for their research participation; overall, the study obtained 514 responses. After removing cases that were incomplete or had response errors, the final sample yielded 440 valid cases. Of these cases, 42.3% reported having received at least one dose of the HPV vaccine; the remaining 57.7% were treated as the unvaccinated group (including those who were not vaccinated or unsure of their vaccination status). The gender split of the sample indicated 57.7% women, 41.1% men, and 1.1% other identity. The racial/ethnic breakdown of the sample is as follows: 70.2% White, 18.0% Asian, 7.5% Black, 11.6% Hispanic/Latino (non-White), 0.7% Native American, and 0.2% Pacific Islander/multiracial. The academic standing of the sample shows 31.6% freshmen, 49.3% sophomores, 9.5% juniors, and 9.5% seniors.

An online experiment utilizing a one-factor between-subjects design (facts-only vs. facts→threat vs. facts→threat→hope) was administered. Upon logging in to the study website and completing the informed consent procedure, participants responded to questions related to demographic characteristics, vaccine perceptions, risk-taking tendencies, and prior exposure to HPV-related messages across various information sources. Following that, participants were randomly assigned to one of three experimental conditions, each with HPV-prevention messages. Afterwards, participants completed the measures for the manipulation check, emotional response toward the message, susceptibility perception, severity perception, vaccine effectiveness perception, and HPV vaccination intentions (for unvaccinated individuals only).

### 3.2. Stimuli and Manipulation Check

Three experimental conditions—facts-only, facts→threat, and facts→threat→hope—were developed to explore how the emotional flow pattern of the message might influence participants’ cognitive and affective response toward HPV and the HPV vaccines. Specifically, the “facts-only” condition describes the basic scientific facts, stating (1) 85% of people will be exposed to the HPV virus without knowing it, unless they are tested or experience symptoms; (2) many infections clear up without causing cancer or other health issues; and (3) the existing HPV vaccines are highly effective in protecting people from developing infections and cancer.

The “facts→threat” condition includes the facts stated above and additional facts intended to increase perceived HPV-related risk by emphasizing uncertainty surrounding immunity. Specifically, this condition highlighted that (1) laboratory research has not established a direct correlation between antibody test and immunity and (2) although HPV vaccines are highly effective, a minimum protective antibody threshold has not been clearly identified. This threat framing aimed to heighten perceived HPV susceptibility and severity.

The “facts→threat→hope” condition presents the factual and threat messaging described above, in addition to a hopeful message stating that (1) more than 98% of vaccinated individuals develop an antibody response after completing the vaccine series and (2) medical scientists and physicians strongly recommend that young people aged 15–26 years get vaccinated immediately (if not yet vaccinated between ages 9 and 12). This message was intended to provide a hopeful feeling to cultivate response efficacy.

After reviewing the experimental message, participants in each study condition were asked to respond to a question, “Did you perceive a shift in the emotional tone of the message?” This was followed by instructing the participants to review the following three statements; each of these statements reflects an emotional tone that matches a message condition. These three statements include (1) “The message maintained a consistent emotional tone throughout,” (2) “The message shifted from a non-emotional tone to a fearful tone,” and (3) “The message shifted from a non-emotional tone to a fearful tone, and then to a hopeful tone.”

Chi-Square tests were conducted to examine whether participants correctly perceived the intended emotional structure of the message to which they were exposed. Results confirmed the consistency in the emotional tone of the facts-only condition, as no significant differences were found in the emotional response among participants, χ^2^(2, *n* = 147) = 1.98, *p* = 0.37. For the facts→threat condition, statistically significant results indicate that emotional tone shift was recognized by a smaller number of participants, χ^2^(2, *n* = 146) = 15.38, *p* < 0.001. The same is true for the facts→threat→hope condition, where the statistically significant findings also suggested that a smaller group of participants detected the emotional tone shift, χ^2^(2, *n* = 147) = 8.88, *p* = 0.012.

### 3.3. Measures

The definition for each construct is described below. A 7-point Likert-type scale, ranging from “strongly disagree” to “strongly agree”, was used to measure all scaled variables, unless otherwise noted. A principal component analysis (with varimax rotation) was applied to all scaled variables to group them into factors that reflect the conceptual dimension(s) for each variable. This was followed by a Cronbach’s α reliability test to evaluate the internal consistency of each conceptual dimension.

HPV message exposure described the exposure to a range of HPV vaccine advertisement or message sources, including (1) out-of-home ads (posters, billboards, signages), (2) television ads, (3) radio/podcast ads, (4) website ads, (5) social media sponsored ads, (6) social media influencers, (7) health experts, and (8) news media reports. These eight items were summed to construct an HPV message exposure index.

Risk-taking tendency assessed perceived likelihood of engaging in various risk behaviors in the future with five items adapted from the Risk-Taking Inventory [46]. These potentially risky behaviors represent a range of scenarios and were clustered into a single factor (α = 0.80). Examples of these items include how often participants planned to engage in “potential recreational risks (e.g., downhill skiing, rock climbing, diving, rollercoaster riding)” and “potential health risks (e.g., consuming raw meat/fish, tobacco smoking, alcohol use, electronic cigarette vaping, energy drink consumption)”.

Vaccine perceptions gauged perceived risks and benefits of the HPV vaccines—by utilizing 10 items adapted from the Vaccine Hesitancy Scale developed by the WHO—which aligned with subsequent psychometric validation work [38,47]. Factor analysis generated two factors, with the first factor reflecting positive vaccine perceptions (e.g., “Vaccines are important for my health”) (α = 0.73), while the second captured perceived safety concerns (e.g., “I am concerned about serious adverse effects of vaccines”) (α = 0.95). After reverse coding the second factor, the two factors were combined to create a conceptually coherent variable; the reliability test yielded very strong inter-item reliability (α = 0.92).

Emotional flow evaluated an individual’s emotional shift while processing the HPV message, with six items adapted from Fitzgerald et al. (e.g., “I felt a series of shifts in my emotions”) [25]. All items loaded on the factor; the internal consistency between items was somewhat low but acceptable (α = 0.77).

Perceived severity, indicating perceived seriousness of the negative health consequences caused by HPV infection, was illustrated by four items adapted from Xie et al. and Warkentin et al. (e.g., “HPV is a serious health risk” and “HPV is a potential danger to my long-term health”) [48,49]. As factor analysis generated a single conceptual dimension, these items combined had a low but acceptable measurement reliability (α = 0.70).

Perceived susceptibility, reflecting feelings of anxiety, fear, and terror associated with HPV infection, was gauged by three items adapted from Xie et al. and Osman et al. (e.g., “I would feel anxious if I contracted HPV”) [48,50]. A single conceptual dimension emerged through factor analysis, with high-scale reliability (α = 0.94).

Perceived vaccine effectiveness, reflecting perceived response efficacy via an evaluation of vaccines as protection against HPV infection, deployed three items adapted from Xie et al. and Warkentin et al. (e.g., “Receiving the full series of the HPV vaccine is an effective way to reduce the risk of HPV-related cancers” and “The HPV vaccine provides long-term protection against HPV-related health risks.”) [48,50]. A resulting single conceptual dimension (generated through factor analysis) demonstrated excellent internal consistency (α = 0.96).

Vaccination intention, depicting intentions to receive the HPV vaccine among the unvaccinated participants, was measured with six items adapted from Xie et al. and Warkentin et al. (e.g., “I will consider consulting a health professional about receiving a vaccine in the next 3 months,” “I intend to receive a vaccine in the next 3 months,” and “I intend to schedule a vaccination appointment within the next 3 months.”) [48,50]. A single factor dimension emerged; the reliability test indicated a strong internal consistency within the factor (α = 0.98).

## 4. Results

### 4.1. Descriptive Statistics and Correlations

Table 1 presents the descriptive statistics for all theoretical constructs, which shows HPV fear had the highest mean at 6.07 (*SD* = 1.14; 7-point scale), followed by perceived vaccine effectiveness (*M* = 5.83, *SD* = 1.16) and HPV susceptibility (*M* = 5.10, *SD* = 1.00). While vaccination intentions (*M* = 4.18, *SD* = 1.62) and vaccine perceptions (*M* = 3.93, *SD* = 0.71) had middling-level means, emotional flow (*M* = 3.18, *SD* = 1.57) and risk-taking tendencies (*M* = 2.47, *SD* = 0.86) had the lowest mean values. The correlations results indicate that the highest correlation was between perceived HPV susceptibility and perceived vaccine effectiveness (0.60). Vaccination intention was significantly related to all other variables.

### 4.2. Research Hypotheses and Questions

H1a and H1b predict that emotional flow intensity will vary by exposure to different message conditions and vaccination status, with the emotional flow level expected to be higher for the facts→threat→hope condition than (a) the facts→threat condition and (b) the facts-only condition, in that order. In addition, H1c speculates that the emotional flow level for the facts→threat condition will outpace the facts-only condition. An ANCOVA was conducted with emotional flow as the dependent variable, and message condition (facts-only, facts→threat, facts→threat→hope) and vaccination status (vaccinated vs. unvaccinated) as between-subjects factors. Pre-exposure factors—future risk-taking and vaccine perceptions—were entered as covariates.

**Figure 1:**
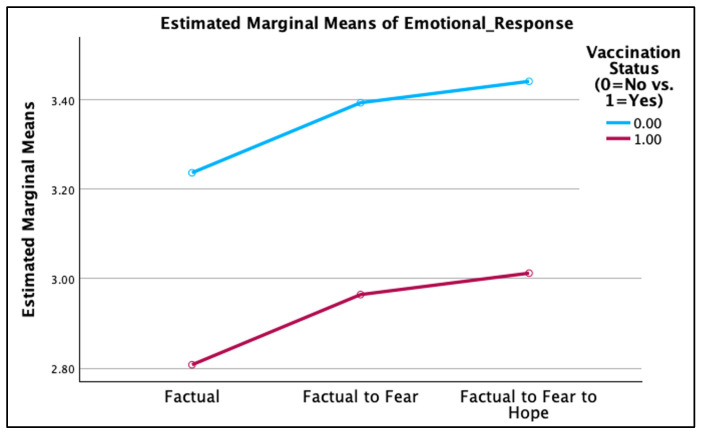
Emotional response between the vaccinated vs. unvaccinated.

Results reported in Table 2 indicate no significant main effect of message condition on emotional flow, *F*(2, 421) = 0.70, *p* = 0.50, partial *η*^2^ = 0.003. Estimated marginal means were similar across conditions (facts-only: *M* = 3.02, *SE* = 0.13; facts→threat: *M* = 3.18, *SE* = 0.13; facts→threat→hope: *M* = 3.23, *SE* = 0.13). Neither the interaction between message condition and vaccination status nor the covariates risk-taking tendencies and vaccine perceptions was a significant predictor of emotional flow. Hence, H1a–H1c were not supported. Vaccination status showed a significant main effect on emotional flow [*F*(1, 421) = 6.18, *p* = 0.01, partial *η*^2^ = 0.01], such that unvaccinated participants reported greater emotional flow than vaccinated participants (see Figure 1 below).

H2a and H2b propose that vaccination intention among unvaccinated individuals will differ due to exposure to different message conditions and will be greater in the facts→threat→hope condition than (a) the facts→threat condition and (b) the facts-only condition, respectively. In addition, H2c asserted that vaccination intention will be greater in the facts→threat condition than the facts-only condition.

An ANCOVA was conducted with the same covariates as in H1a–H1c. No significant effect of message condition was observed, *F*(2, 236) = 1.31, *p* = 0.272, partial *η*^2^ = 0.011 (see Table 3). Therefore, H2a–c were not confirmed. Both covariates, risk-taking tendencies and vaccine perceptions, were significant predictors of vaccination intentions.

H3a–H3d separately compared the level of (a) emotional flow, (b) perceived HPV susceptibility, (c) perceived HPV severity, and (d) perceived vaccine effectiveness between vaccinated and unvaccinated individuals across the three study conditions, respectively. An independent-samples *t*-test indicated that unvaccinated participants reported significantly higher emotional flow than vaccinated participants in the facts→threat→ hope condition, *t*(144) = 2.95, *p* = 0.004, Cohen’s *d* = 0.49 (see Table 4). No significant differences were observed in either the facts-only or facts→threat conditions. Thus, H3a was not supported.

As for H3b, the *t*-test indicated that vaccinated participants reported significantly greater HPV susceptibility in—(1) the facts-only condition [*t*(144.07) = 3.43, *p* < 0.001, Cohen’s *d* = 0.54], (2) the facts→threat condition [*t*(145) = 2.19, *p* = 0.03, Cohen’s *d* = 0.36], and (3) the facts→threat→hope condition [*t*(137.15) = 3.56, *p* < 0.001, Cohen’s *d* = 0.56]—than unvaccinated participants (see Table 4). These findings validated H3b.

Turning to H3c, the *t*-test showed that vaccinated participants reported significantly higher perceptions of HPV severity than unvaccinated participants in the facts-only condition [*t*(144) = 2.92, *p* = 0.004, Cohen’s *d* = 0.49] and the facts→threat condition [*t*(145) = 2.01, *p* = 0.046, Cohen’s *d* = 0.33]. In contrast, no significant difference in perceived HPV severity was observed between vaccinated and unvaccinated participants in the facts→threat→hope condition, *t*(144) = 0.53, *p* = 0.597, Cohen’s *d* = 0.09. These results provided partial confirmation of H3c (see Table 4).

In terms of H3d, the *t*-test revealed that vaccinated participants consistently reported significantly greater perceived vaccine effectiveness in the facts-only condition [*t*(143.13) = 6.09, *p* < 0.001, Cohen’s *d* = 0.97], the facts→threat condition [*t*(144) = 3.54, *p* < 0.001, Cohen’s *d* = 0.59], and the facts→threat→hope condition [*t*(143.79) = 4.46, *p* < 0.001, Cohen’s *d* = 0.71]. These findings supported H3d.

RQ1a,b examined whether vaccinated participants would report higher pre-exposure (a) risk-taking tendencies and (b) vaccine perceptions, respectively, when compared to unvaccinated participants. Independent-samples *t*-test results indicated a significant difference in vaccine perceptions in the facts→threat→hope condition, with vaccinated individuals (*M* = 5.65, SD = 0.99) rating them higher than unvaccinated individuals [(*M* = 5.10, SD = 1.11), *t*(438) = 5.40, *p* < 0.001, Cohen’s *d* = 0.52]. However, risk-taking tendencies did not significantly differ between vaccinated and unvaccinated participants.

RQ2 queries whether the exposure level of HPV messages across various information sources will differ between vaccinated and unvaccinated individuals. An independent-samples *t*-test indicated both groups of participants were exposed to a similar number of HPV messages across the eight information sources presented to them.

RQ3 inquires whether exposure to the different HPV information sources is associated with cognitive and affective responses to HPV and the HPV vaccines. Table 5 describes the zero-order correlation analysis results, which suggest that (1) vaccine perceptions were negatively correlated with exposure to HPV radio ads (*r* = −0.10, *p* = 0.05) and social media influencers (*r* = −0.13, *p* ≤ 0.01) and (2) emotional response to the study stimuli was positively related to exposure to social media ads (*r* = 0.11, *p* ≤ 0.05), web-based ads (*r* = 0.11, *p* ≤ 0.05), and health experts (*r* = 0.17, *p* ≤ 0.01).

While HPV susceptibility was negatively and positively linked to exposure to social media influencer (*r* = −0.16, *p* ≤ 0.01) and health expert messages (*r* = 0.15, *p* ≤ 0.01), respectively, HPV severity was positively correlated with exposure to both outdoor ads and health expert messages. As perceived vaccine effectiveness was negatively connected to exposure to social media influencer and positively related to health expert messages, vaccination intention was found to be irrelevant to the exposure to any of the eight HPV message sources.

Additional descriptive analysis of the frequency of exposure to the eight HPV message categories demonstrated that the most frequently cited source was television ads (41.8%), followed by social media-sponsored ads (24.1%) and health experts/professionals (20.5%). These are followed by exposure to out-of-home ads (14.3%) and website ads (14.1%). The least heard or seen HPV message sources include news reports (8.4%), social media influencers (4.5%), and radio/podcast ads (1.4%). Total percentages reported here exceed 100% because participants could select multiple sources.

An additional analysis was conducted using a hierarchical regression to explore the potential predictors of vaccination intentions among unvaccinated participants. The order of predictor entry is as follows: (1) Step 1: risk-taking tendencies and vaccine perceptions, (2) Step 2: emotional flow, (3) Step 3: perceived HPV susceptibility and severity, and (4) Step 4: perceived vaccination effectiveness. As demonstrated in Table 6, the final model indicated perceived HPV susceptibility and vaccine effectiveness failed to predict vaccination intentions. By contrast, risk-taking tendencies (β = 0.20, *p* < 0.001), vaccine perceptions (β = 0.31, *p* < 0.001), emotional flow (β = 0.19, *p* = 0.047), and perceived HPV severity (β = 0.22, *p* = 0.0030) were significant predictors of vaccination intentions. As the highest VIF value observed was 2.388, no multicollinearity concern was detected. The overall equation explained 26% of the total variance in the criterion variable.

## 5. Discussion

The current study tested whether a health protection message promoting HPV vaccination designed with an emphasis on “HPV facts” vs. “HPV facts and threat” vs. “HPV facts combined with threat and hope” may differentially influence individuals’ emotional response toward HPV and vaccination intention, in addition to other related cognitive and affective outcomes. As past studies addressing the emotional flow construct included no more than two message conditions (e.g., fear and/or fear-to-efficacy), our study provided a baseline condition (facts only) to provide a more ecological representation of the HPV prevention messages seen by the public. We also adopted a novel approach by comparing vaccinated vs. unvaccinated individuals to compare their responses to our message design, as well as exploring the association between exposure to the key HPV message sources with HPV vaccine beliefs and attitude measures.

Contrary to expectations, our results revealed that participants did not indicate a significant emotional shift due to exposure to messages that incorporated an emotional tone shift. The two pre-exposure covariates—risk-taking tendencies and vaccine perceptions—were also irrelevant predictors of emotional shift. Hence, the experiment failed to generate a significant difference in emotional response across the three different HPV prevention messages.

Nonetheless, the ANCOVA and post hoc *t*-tests results revealed that vaccinated individuals showed a significantly greater perceived HPV susceptibility, HPV severity, and perceived vaccine effectiveness across all three study conditions (i.e., facts-only, facts→threat, and facts→threat→hope) than unvaccinated individuals. These results are consistent with prior research, which similarly reported how vaccinated individuals scored higher than their unvaccinated counterparts in perceived HPV vulnerability and HPV severity, as well as vaccine perceptions/benefits and perceived vaccine effectiveness [32,40]. They also confirm previous findings that established that individuals who had a more positive appraisal of vaccination benefits also expressed greater hope (or efficacy) associated with vaccination [21,42].

These results suggest that vaccination status might have helped shape how individuals processed the HPV messages. Specifically, the three prevention messages appeared to be more effective in eliciting a response toward the knowledge (HPV facts), threat appraisal (HPV risk), and/or response efficacy (vaccine effectiveness) among vaccinated instead of unvaccinated individuals. This interpretation is logical because vaccinated individuals are more aware of the HPV risk and prevention actions than their unvaccinated counterparts [27,28,29,30,31].

In addition, the ANCOVA and the post hoc *t*-tests results also demonstrated that vaccination status had a significant effect on emotional-shift response—with unvaccinated individuals reporting a greater level of emotional shift than vaccinated individuals—in the facts→threat→hope condition. By implication, the particular message design, which provided the “facts” for education, the “threat” to cause fear, and “hope” to build efficacy, seemed to be more appealing to unvaccinated than vaccinated individuals. This probable explanation stems from the fact that vaccinated individuals are more familiar with HPV risks and prevention actions and may be less likely to react to the message emotionally [27,28,29,30,31].

Taken together, our findings imply that as unvaccinated individuals demonstrated an emotional shift toward the HPV message that incorporates hopeful or efficacy-related cues, this pattern aligns with previous studies examining threat-based messaging and emotional shifts in risk communication contexts [18,25,26]. This implication is similarly consistent with prior work suggesting that combining threat- and efficacy-related cues may be salient for vaccine-hesitant populations [34]. It also echoes the work of Carcioppolo et al., which discovered a one-to-one threat-to-efficacy ratio was the most effective strategy to increase HPV vaccination intention among young women [35].

Importantly, it is worth noting that emotional flow manipulations alone may not be sufficient to generate the intended effect to bridge the attitude–intention gap. Other factors not measured here—such as vaccination cost, vaccine access, social stigma, and recommendation from a trusted source—could also contribute to this gap. Importantly, these results underscore the need for caution—when inferring persuasive effectiveness from emotional responses elicited from messages that include fear and efficacy—among those who remain unvaccinated.

Turing to the regression results, vaccine perception was the strongest significant predictor of vaccination intention, followed by HPV severity, risk-taking tendencies, and emotional response. Based on these results, both cognitive and affective factors are at play, as individuals weigh the benefits of vaccines and HPV threat to determine whether to accept the vaccination risk, alongside their shifting emotions. These results align with the factors identified in past research—such as perceived severity of HPV [51], sensation seeking, risk-taking behaviors, and neutral [52] and positive attitudes toward the vaccines [53]—that can influence an individual’s decision to accept vaccination and the potential risk associated with it [19,32,37].

Contrastingly, HPV susceptibility and vaccine effectiveness were not found to be significant predictors of vaccination intention. Hence, awareness of one’s risk to a virus and the efficacy of vaccines as a protective measure do not necessarily lead to vaccine uptake. These results are similar to those evidenced by Chen’s study, which discovered that perceived COVID-19 vulnerability (operationalized as fear in the current study) was a negative predictor and perceived vaccine effectiveness was a non-significant predictor of vaccine acceptance [53].

As for the pattern that emerges from exposure to HPV prevention campaign ads, no significant difference was found between the vaccinated and unvaccinated individuals. By looking into the one-on-one correlation between each HPV messaging source and HPV- and vaccination-related factors, it is clear that health experts as well as ads appearing on social media platforms and the Internet in general had a weak-but-positive association with emotional response toward the HPV prevention messages tested in the study. While health expert recommendations were positively linked to HPV fear, the opposite was true for the social media ads. Furthermore, as messages from health experts were linked to greater HPV fear, the opposite was true for HPV prevention ads appearing in social media. It should be noted that the interpretation of the correlations provided here is purely descriptive instead of inferential in nature.

Additional descriptive statistics indicated that at least one out of five study participants was exposed to TV ads (41.8%), social media ads (24.1%), and health experts (20.1%) that promoted HPV prevention. This suggests that health campaigns may consider focusing on creating HPV prevention messages in the format of TV and social media ads, as well as casting health experts to appear in these ads, to reach Gen Z young adults. These results are similar to those reported by Chen et al., which reported that the communication medium chosen to deliver the HPV prevention message (e.g., television, online ads, social media platforms) could influence message recall and attitudes toward vaccination [9].

According to Laily et al., 79.1% of the HPV-aware adults (*N* = 2214) surveyed considered “some” to “a lot” of the HPV information seen on social media to be inaccurate [39]. Hence, in order to encourage the audience to get vaccinated, the HPV prevention ads must be seen as trustworthy and provide sufficient motivation for prompting vaccination action. This means that the substance of both the TV and social media ads needs to convey the correct facts, in addition to presenting an adequate fear-to-hope (efficacy) ratio to promote knowledge gain and an emotional connection in the audience.

Several study limitations should be noted when interpreting the current findings. First, the U.S. college student sample reduces generalizability to the broader Gen Z population. Future research should include non-college-educated young adults in the sample. Second, the use of a one-shot online experiment, as well as self-reported vaccination status and psychometric measures, may limit measurement validity. Employing a field experiment with a repeated-measure design will enhance the ecological validity and allow the assessment of attitude–behavior gap changes over time. Third, the small effect sizes observed for emotional tone shifts suggest that additional message elements—such as explicit instructions about vaccine adoption recommended by trusted sources (e.g., healthcare professionals) and access to vaccination services—could be added to more explicitly reflect the construct of “hope” or response efficacy. Finally, future studies should include more racially and ethnically diverse samples and more naturalistic designs to evaluate whether emotional responses translate into sustained vaccination behavior.

## 6. Conclusions

This research contributes to both theoretical and practical implications. Theoretically, it expands the application of EPPM by integrating the construct of emotional flow to address its static treatment of fear and efficacy. Practically, it offers insights for practitioners to understand the cognitive and affective barriers to HPV vaccine adoption. Although messages incorporating emotional flow were associated with greater emotional response intensity more among unvaccinated than vaccinated individuals, they did not lead to significantly higher vaccination intentions, compared to the baseline facts-only control message.

Vaccination status emerged as a key factor shaping both pre- and post-exposure HPV risk- and vaccine-related perceptions. Vaccinated participants reported greater perceived benefits of the HPV vaccine, whereas unvaccinated participants exhibited stronger emotional reactions toward the emotional flow message about vaccination. This suggests that while emotional tone can influence selected cognitive and affective responses, it may not be sufficient to drive behavioral intention or bridge the attitude–behavior gap. As such, emphasizing the facts (e.g., medical evidence), threat (e.g., the severity of cancer risks), and hope (e.g., vaccine efficacy and access) may enhance message effectiveness.

## Figures and Tables

**Table 1:** Descriptive statistics and zero-order correlations.

	1	2	3	4	5	6	7
1 Future Risk-Taking	--	--					
2 Vaccine Perceptions	−0.09	--					
3 Emotional Response	0.06	−0.01 *	--				
4 HPV Susceptibility	0.08	0.35 **	−0.03	--			
5 HPV Severity	0.17 **	0.24 **	0.13 **	0.47 **	--		
6 Vaccine Effectiveness	0.06	0.62 **	−0.07	0.60 **	0.49 **	1	
7 Vaccination Intention	0.27 **	0.33 **	0.22 **	0.19 **	0.35 **	0.33 **	1
Mean	2.47	3.93	3.18	6.07	5.10	5.83	4.18
Standard Deviation	0.86	0.71	1.57	1.14	1.00	1.16	1.62

* *p* < 0.05 (2-tailed); ** *p* < 0.01 (2-tailed).

**Table 2:** ANCOVA results for post-exposure emotional response.

Source	*F*	*p*	*η* ^2^
Corrected Model	2.23	0.03	0.04
Message Condition	0.70	0.50	0.003
Vaccinated vs. Unvaccinated	6.18	0.01	0.01
Message Condition × Vaccination Status	1.24	0.29	0.01
Future Risk-Taking	1.42	0.23	0.003
Vaccine Perceptions	1.75	0.19	0.004

Note: *η*^2^ values represent partial eta squared.

**Table 3:** ANCOVA results for post-exposure vaccination intention (among unvaccinated participants).

Source	*F*	*p*	*η* ^2^
Corrected Model	14.00	<0.001	0.19
Three Message Conditions	1.38	0.25	0.01
Future Risk-Taking	20.32	<0.001	0.08
Vaccine Perceptions	36.62	<0.001	0.13

Note: *η*^2^ values represent partial eta squared.

**Table 4:** T-test results for post-exposure measures between the vaccinated and unvaccinated.

		Facts-Only		Facts→Threat		Facts→Threat→Hope	
		Vaccinated	Unvaccinated	Vaccinated	Unvaccinated	Vaccinated	Unvaccinated
Emotional Flow	*M*	2.82	3.25	3.12	3.29	2.83 **	3.57
	*SD*	1.65	1.54	1.67	1.49	1.52	1.50
HPV Susceptibility	*M*	6.43 ***	5.78	6.26 *	5.85	6.47 ***	5.91
	*SD*	0.96	1.32	1.05	1.17	0.71	1.18
HPV Severity	*M*	5.36 **	4.86	5.36 *	5.04	5.27	5.18
	*SD*	1.03	1.04	0.90	1.00	0.87	1.02
Vaccine Effectiveness	*M*	6.38 ***	5.39	6.24 ***	5.65	6.30 ***	5.43
	*SD*	0.85	1.13	0.87	1.12	0.99	1.35

Note: * *p* < 0.05; ** *p*< 0.01; *** *p* < 0.001.

**Table 5:** Correlations between HPV message sources, vaccine beliefs, and HPV emotions.

	Out-of- Home Ads	Radio Ads	TV Ads	Social Media Ads	Web Ads	Influencer Message	Health Expert Messages	News Reports
Vaccine Perceptions	−0.02	−0.10 *	−0.07	−0.06	−0.09	−0.13 **	0.04	0.004
Emotional Flow	−0.01	−0.04	0.08	0.11 *	0.11 *	0.05	0.12 *	0.04
HPV Susceptibility	0.03	−0.03	0.002	0.01	0.01	−0.16 **	0.15 **	−0.002
HPV Severity	0.09 *	0.02	0.05	0.01	0.05	−0.01	0.10 *	−0.02
Vaccine Effectiveness	0.04	−0.04	0.03	−0.02	−0.08	−0.10 *	0.12 *	−0.004
Vaccination Intention	−0.02	−0.05	−0.11	0.001	−0.02	−0.02	0.11	0.04

* *p* < 0.05 (2-tailed); ** *p* < 0.01 (2-tailed).

**Table 6:** Regression results for vaccination intention (among the unvaccinated).

		β	T	*p*	VIF	Adjusted R^2^	*p*
Model							
1	Risk-Taking Tendencies	0.26	4.33	<0.001	1.012	0.178	<0.001
	Vaccine Perceptions	0.37	6.30	<0.001	1.012		
2	Risk-Taking Tendencies	0.24	4.23	<0.001	1.016	0.053	<0.001
	Vaccine Perceptions	0.38	6.58	<0.001	1.015		
	Emotional Flow	0.23	4.06	<0.001	1.006		
3	Risk-Taking Tendencies	0.20	3.57	<0.001	1.052	0.041	
	Vaccine Perceptions	0.34	5.77	<0.001	1.141		0.002
	Emotional Flow	0.19	3.25	<0.001	1.058		
	HPV Susceptibility	−0.07	−1.03	0.306	1.527		
	HPV Severity	0.24	3.52	<0.001	1.542		
4	Risk-Taking Tendencies	0.20	3.51	<0.001	1.057	0.002	0.428
	Vaccine Perceptions	0.31	4.37	<0.001	1.641		
	Emotional Flow	0.19	3.28	0.001	1.061		
	HPV Susceptibility	−0.09	−1.22	0.222	1.695		
	HPV Severity	0.22	3.04	0.003	1.741		
	Vaccine Effectiveness	0.07	0.79	0.428	2.381		

## Data Availability

The data are available upon request.
